# Successful treatment of schizophrenia with melperone augmentation in a patient with phenotypic CYP2D6 ultrarapid metabolization: a case report

**DOI:** 10.1186/1752-1947-6-49

**Published:** 2012-02-06

**Authors:** Maximilian Gahr, Regina Gastl, Markus A Kölle, Carlos Schönfeldt-Lecuona, Roland W Freudenmann

**Affiliations:** 1 Department of Psychiatry and Psychotherapy III, University of Ulm, Ulm, Germany; 2 Department of Neurology, University of Ulm, Ulm, Germany

## Abstract

**Introduction:**

There are limited treatment options for people with schizophrenia with cytochrome P450 2D6 ultrarapid metabolizer status who do not respond to amisulpride.

Furthermore, the literature does not provide evidence-based guidelines for this particular constellation.

**Case presentation:**

We report the case of a 50-year-old Caucasian female patient with schizophrenia and cytochrome P450 2D6 ultrarapid metabolizer status who experienced an insufficient antipsychotic effect with amisulpride. She was successfully treated with melperone-augmented haloperidol.

**Conclusion:**

This report yields melperone-augmented haloperidol as a possible pharmacological strategy in the described situation. In addition, our observations support the available evidence for the potential of melperone to act as an inhibitor of cytochrome P450 2D6.

## Introduction

Enhancing the effect of a pharmacological agent by adding on another drug is commonly called augmentation. One possible augmentation strategy is via induction of pharmacokinetic interactions. Here the added agent interacts with enzymes of the cytochrome P450 system (CYP) that is important for the hepatic metabolization of numerous drugs. Thus, utilizing the pharmacokinetic properties of a particular agent allows its application as a specific inhibitor or inductor of metabolic pathways of the drug to be enhanced. This can, for example, allow decreased doses to result in sufficient plasma level concentrations. Within the CYP family, CYP2D6 is a polymorphic enzyme that is of relevance for the metabolism of most typical antipsychotics [1]. There are more than 100 genetic variants (catalogued by the website: http://www.cypalleles.ki.se). Out of these, there are four phenotypes (poor, intermediate, extensive and ultrarapid metabolizers) that are associated with different enzyme activities and whose incidence vary among ethnic groups [2]. Ultrarapid metabolizers (UM) are people with markedly elevated enzyme activity; there are estimated to be between 0.5% and 7% UMs within the Caucasian population [3,4]. Patients with intractable schizophrenia and CYP2D6 UM status can benefit from a treatment with the antipsychotic amisulpride, which is almost completely eliminated through the renal pathway. Therapeutic alternatives for non-responders under amisulpride are limited and evidence-based guidelines regarding this particular treatment situation are lacking. In the following, we report successful treatment with melperone-augmented haloperidol in a patient with schizophrenia and UM status.

## Case presentation

Our patient was a 50-year-old Caucasian woman, a non-smoker, with a 20-year history of undifferentiated schizophrenia, according to the Diagnostic and Statistical Manual, Fourth Edition, Text Revision criteria. She was admitted to our clinic for acute psychotic exacerbation. Due to numerous treatments in our clinic over the last 10 years, our patient was well known and the diagnosis of schizophrenia had been secured based on several psychotic episodes and unremarkable somatic examination, including analysis of her cerebrospinal fluid, an electroencephalogram and magnetic resonance imaging of her brain.

Initially, she presented with auditory hallucinations, disorganized speech, inappropriate affect with an occasionally euphoric mood, grossly disorganized behavior and excitability. Prior to this hospital admission, her antipsychotic treatment (retard preparation of quetiapine, 700 mg per day) was taken irregularly. Further, impaired glucose tolerance due to adiposity and metabolic syndrome (weight 102.8 kg, height 156 cm, body mass index 42.2 kg/m^2^) was treated with metformin (500 mg per day). We first continued quetiapine for seven days without amelioration of the clinical situation. In view of sufficient quetiapine serum levels (854 ng/mL) and remarkable obesity, a treatment trial with an increased dosage of quetiapine was not performed. While tapering off quetiapine, we administered amisulpride and titrated up to a daily dosage of 1200 mg within five days. No substantial improvement was achieved with this regimen within 21 days.

Phenotypic testing in our patient with dextromethorphan (DM) revealed CYP2D6 UM status (DM < 5 ng/mL; dextrorphan (DO) 640 ng/mL; DM/DO ratio: < 0.008; 3-methoxymorphinan (MM) < 5 ng/mL; 3-hydroxymorphinan (HM) 24 ng/mL; MM/HM ratio: 0.21), which also explained recent ineffective treatment attempts with risperidone (up to 8 mg per day) and haloperidol-decanoate (up to 150 mg per injection). This phenotypic testing was performed by taking a blood sample (120 mL) exactly one hour after the oral application of 40 mL NeoTussan^® ^cough syrup (111 mg dextromethorphan per 100 g suspension (Novartis Consumer Health GmbH; city: Munich; state: Bavaria; country: Germany). Her blood was analyzed for metabolites of DM and MM, which are substrates of CYP2D6. The DM to DO and MM to HM ratios are surrogate parameters for CYP2D6 activity.

Following the reduction of amisulpride down to 800 mg per day, haloperidol (10 mg per day) was applied as an additional antipsychotic. Simultaneously, trazodone (100 mg per day) was added on in order to augment the haloperidol by CYP2D6 inhibition, but did not show significant clinical success in the next days. Gradual escalation of haloperidol and trazodone doses over a period of one month did not obtain amelioration of the psychotic phenomena. At that time, haloperidol plasma levels (current medication: amisulpride 800 mg per day; haloperidol 15 mg per day; trazodone 200 mg per day; and metformin 500 mg per day) were at 10 ng/mL. A subsequent increase of haloperidol up to 20 mg per day did neither improve the clinical situation nor serum plasma levels (9 ng/mL). Since her psychopathological findings did not change under treatment with amisulpride and still presented as on admission time, the antipsychotic effect of amisulpride was evaluated to be insufficient according to the treatment guideline for schizophrenia of the German Association for Psychiatry, Psychotherapy and Neuroscience [5]. After discontinuation of trazodone and amisulpride, we administered melperone (100 mg per day), in order to inhibit CYP2D6, and elevated haloperidol up to 30 mg per day. After 14 days, her haloperidol plasma levels were at 19 ng/mL. This result was confirmed in a second test (haloperidol: 20 ng/mL). In line with this, her clinical performance improved slightly, and she demonstrated attenuation in her auditory hallucinations, redevelopment of day structure and a marked improvement regarding behavior and speech. Our patient was then discharged with this medication in a clearly improved clinical condition.

## Discussion

There are no evidence-based guidelines in the literature for patients with schizophrenia and CYP2D6 UM status that do not respond to amisulpride. Our report yields the combination of a high dose of haloperidol augmented with melperone as a possible pharmacological strategy in patients under these conditions. Considering that the patient´s haloperidol serum levels increased concurrently with elevated doses of haloperidol, the question arises to what the melperone effectively contributed to sufficient haloperidol serum levels. Though reports on the potential of melperone as an inhibitor of CYP2D6-dependent metabolization of psychopharmacologic drugs are available [6,7], it is conceivable that, in our case, sufficient haloperidol plasma levels were simply a consequence of escalated oral haloperidol doses, and to a lesser extent induced by melperone-mediated CYP2D6 inhibition. Discontinuation of melperone and repeated measurements of haloperidol serum levels under the removed influence of melperone could have provided clarity. However, due to the unstable clinical situation and first-time treatment success with melperone-augmented haloperidol, we refrained from any modification of the psychopharmacotherapy. Apart from that, the combination of haloperidol and melperone was successful in our patient with regard to haloperidol plasma levels and schizophrenic target symptoms. Retrospectively, the repeated modifications of psychopharmacotherapy in this case during a comparatively short period of time are problematic and thus, for example, continuation of amisulpride for a longer interval should have been attempted.

## Conclusion

We hypothesize that melperone-augmented haloperidol can be considered as a possible treatment strategy in patients with schizophrenia, CYP2D6 UM status and insufficient antipsychotic effect of amisulpride. Furthermore, our observation of sufficient haloperidol plasma levels under augmentation with melperone is in line with studies that described melperone as an inhibitor of CYP2D6-dependent metabolization of risperidone [6] and venlafaxine [7].

## Consent

Written informed consent was obtained from the patient's legal guardian for publication of this case report. A copy of the written consent is available for review by the Editor-in-Chief of this journal.

## Competing interests

The authors declare that they have no competing interests.

## Authors' contributions

MG wrote the manuscript. RG, MMK and CSL were involved in the patient's treatment and investigation of data. RWF was a major contributor in writing the manuscript. All authors read and approved the final manuscript.
